# Semi-MsST-GAN: A Semi-Supervised Segmentation Method for Corneal Ulcer Segmentation in Slit-Lamp Images

**DOI:** 10.3389/fnins.2021.793377

**Published:** 2022-01-04

**Authors:** Tingting Wang, Meng Wang, Weifang Zhu, Lianyu Wang, Zhongyue Chen, Yuanyuan Peng, Fei Shi, Yi Zhou, Chenpu Yao, Xinjian Chen

**Affiliations:** ^1^Medical Image Processing, Analysis and Visualization (MIPAV) Laboratory, The School of Electronics and Information Engineering, Soochow University, Suzhou, China; ^2^The State Key Laboratory of Radiation Medicine and Protection, Soochow University, Suzhou, China

**Keywords:** corneal ulcer, GAN, slit-lamp image, semi-supervision, deep learning

## Abstract

Corneal ulcer is a common leading cause of corneal blindness. It is difficult to accurately segment corneal ulcers due to the following problems: large differences in the pathological shapes between point-flaky and flaky corneal ulcers, blurred boundary, noise interference, and the lack of sufficient slit-lamp images with ground truth. To address these problems, in this paper, we proposed a novel semi-supervised multi-scale self-transformer generative adversarial network (Semi-MsST-GAN) that can leverage unlabeled images to improve the performance of corneal ulcer segmentation in fluorescein staining of slit-lamp images. Firstly, to improve the performance of segmenting the corneal ulcer regions with complex pathological features, we proposed a novel multi-scale self-transformer network (MsSTNet) as the MsST-GAN generator, which can guide the model to aggregate the low-level weak semantic features with the high-level strong semantic information and adaptively learn the spatial correlation in feature maps. Then, to further improve the segmentation performance by leveraging unlabeled data, the semi-supervised approach based on the proposed MsST-GAN was explored to solve the problem of the lack of slit-lamp images with corresponding ground truth. The proposed Semi-MsST-GAN was comprehensively evaluated on the public SUSTech-SYSU dataset, which contains 354 labeled and 358 unlabeled fluorescein staining slit-lamp images. The results showed that, compared with other state-of-the-art methods, our proposed method achieves better performance with comparable efficiency.

## Introduction

The cornea is a transparent membrane located at the front of the eyeball and is directly exposed to the air. Therefore, it is more likely to be infected with bacteria, resulting in several frequently occurring ophthalmic symptoms such as corneal ulcer. Corneal ulcer is an inflammatory or, more seriously, infective condition of the cornea involving disruption of its stromal–epithelial layers (Bron et al., 2007; Chen and Yuan, 2010). Late or inappropriate treatment may induce irreversible damages to vision acuity (Cohen et al., 1987; Diamond et al., 1999).

Fluorescein staining is the most widely used diagnostic technology in optometry and ophthalmology to assess the integrity of the ocular surface, particularly the integrity of the cornea (Morgan and Carole, 2009; Zhang et al., 2018). With the development of staining techniques, doctors can quantitatively evaluate the size and severity of corneal ulcers by fluorescein staining of slit-lamp images.

**FIGURE 1:**
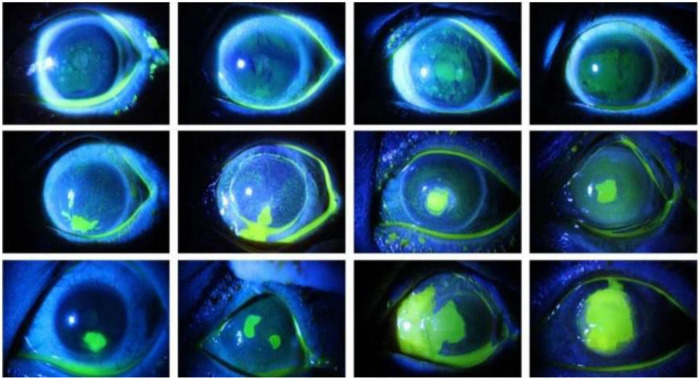
Comparison of the three types of corneal ulcers, with the *top row* representing point-like corneal ulcers, the *middle row* representing point-flaky mixed corneal ulcers, and the *bottom row* representing flaky corneal ulcers.

Accurate segmentation of the ulcer region is essential for assessing the severity of corneal ulcer and formulating a treatment plan. As shown in Figure 1, corneal ulcer can be classified into point-like corneal ulcer, point-flaky mixed corneal ulcer, and flaky corneal ulcer according to the pathological characteristics and distribution. Although the ulcer region can be marked manually by experienced ophthalmologists *via* some professional software, this task is time-consuming and subjective. Therefore, it is significant to explore a method that can automatically and accurately segment the corneal ulcer area.

There are some segmentation methods (Pritchard et al., 2003; Wolffsohn and Purslow, 2003; Peterson and Wolffsohn, 2009) designed for separate point-like corneal ulcers rather than for the point-flaky or flaky types. Later, methods for the segmentation of corneal ulcers with more complex shapes were proposed and achieved good results (Chun et al., 2014; Sun et al., 2017; Deng et al., 2018a,b; Liu et al., 2019). Chun et al. (2014) proposed an objective digital image analysis system to evaluate the corneal staining using RGB (red–green–blue) and the hue–saturation–value (HSV) technique with 100 images. Deng et al. (2018a) presented an automatic ulcer segmentation method by utilizing *k*-means clustering followed by morphological operations and region growing. Then, in Deng et al. (2018b), a simple linear iterative clustering (SLIC) super-pixel-based pipeline was proposed for automatic flaky corneal ulcer area extraction with 150 images. Liu et al. (2019) segmented the ulcer area by employing a joint method of Otsu and Gaussian mixture model (GMM) with 150 images. Sun et al. (2017) proposed a patch-based deep convolutional neural network (CNN) for corneal ulcer segmentation with 48 images. The methods mentioned above are traditional algorithms mostly based on around 100 images and are only designed for certain types of corneal ulcer, therefore not suitable for all types of segmentation.

Recently, several CNNs have been proposed for medical image segmentation, such as UNet (Ronneberger et al., 2015), CE-Net (Gu et al., 2019), Att-UNet (Oktay et al., 2018), and CPFNet (Feng et al., 2020). Most of them are based on the encoder–decoder architecture (Ronneberger et al., 2015) due to its good performance. The encoder can extract the context information and reduce the spatial dimension of feature maps. The decoder can recover the spatial dimension and details of the targets. The skip connections help to recover the full spatial resolution at the network output, making the network suitable for semantic segmentation (Zhou et al., 2018). However, the original skip connections in the U-shaped network will introduce irrelevant clutters and have semantic gaps due to the mismatch of the receptive fields (Feng et al., 2020). To improve the performance of the original U-Net, methods such as attention U-Net (Att-UNet) (Oktay et al., 2018) and CPFNet (Feng et al., 2020) have introduced an attention mechanism, whose core idea is to change the global focus to key and local region focus. The attention mechanism tries to focus the attention of the network on the relationship of the channels, gather spatial information to focus on the correlated features, and suppress the irrelevant regions in the feature map. It is beneficial to utilize attention mechanism to capture more rich details of objects instead of the direct concatenation of feature maps from the encoder and decoder. Although these CNN-based methods have achieved good performance (Ronneberger et al., 2015; Oktay et al., 2018; Gu et al., 2019; Feng et al., 2020), a few CNN-based methods have been proposed for corneal ulcer segmentation in slit-lamp images. There are still two problems that need to be solved in order to improve the accuracy of corneal ulcer segmentation in slit-lamp images: (1) the interferences caused by complicated pathological features of corneal ulcers in slit-lamp images, such as the large differences in the pathological shapes between point-like, point-flaky, and flaky corneal ulcers, blurred boundary, and noise interference, and (2) how to leverage the large amount of unlabeled data to further improve the segmentation accuracy. In this paper, we propose a novel semi-supervised algorithm based on adversarial learning to solve the current dilemma. Our main contributions are summarized as follows:

(1) To improve the segmentation performance of the corneal ulcer regions with complex pathological features, a novel multi-scale self-transformer network (MsSTNet) is proposed for corneal ulcer segmentation, which can improve the ability of the model to capture the global long-range dependencies of multi-scale features from different layers.
(2) To leverage unlabeled samples for the further performance improvement, a novel semi-supervised multi-scale self-transformer generative adversarial network (Semi-MsST-GAN) is explored.
(3) Comprehensive experiments based on the SUSTech-SYSU dataset have been conducted to demonstrate the effectiveness of our proposed methods. The results show that, compared with other state-of-the-art algorithms, our proposed method not only achieves higher segmentation accuracy but also can leverage unlabeled data to further improve segmentation performance.

## Methods

We adopted the adversarial framework as the architecture of our proposed method, which contains a generator network and a discriminator referred to Mirza and Osindero (2014) and Isola et al. (2017). The following provides a detailed description and functional interpretation of the proposed method.

### Semi-MsST-GAN

In recent years, generative adversarial networks (GANs) (Goodfellow et al., 2014) and their variations (Chen et al., 2016; Ma et al., 2018; Wang T.-C. et al., 2018; Jiang et al., 2019) have been widely used in several domains (Li and Wand, 2016; Pathak et al., 2016; Salimans et al., 2016; Vondrick et al., 2016; Wu et al., 2016; Zhu et al., 2016, 2017; Zha et al., 2019), especially in image processing applications, such as image generation (Zha et al., 2019), image editing (Zhu et al., 2016), representation learning (Salimans et al., 2016), image inpainting (Pathak et al., 2016), style transfer (Li and Wand, 2016), and image-to-image translation (Zhu et al., 2017), with significant performances. Different from the original GAN that generates images based on random noise, conditional GAN (cGAN) generates images based on specified conditional inputs (Mirza and Osindero, 2014). Moreover, the GAN architecture is also widely used in semi-supervision-based methods (Sricharan et al., 2017; Hung et al., 2018; Wang et al., 2021). Therefore, to improve the ability of the model to learn the complex pathological features and leverage unlabeled data in order to further improve the segmentation performance, we proposed a novel semi-supervised MsST-GAN based on cGAN architecture for corneal ulcer segmentation.

**FIGURE 2:**
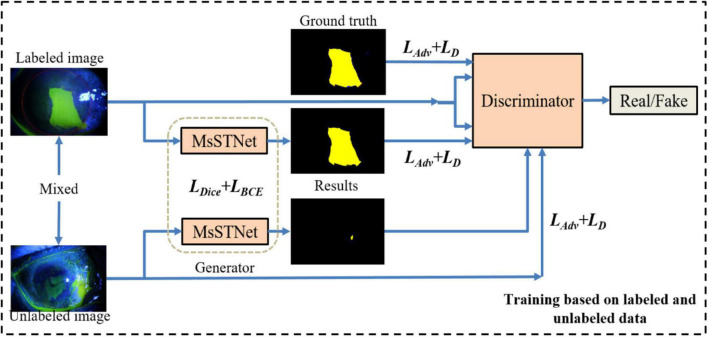
Framework of the proposed semi-supervised multi-scale self-transformer generative adversarial network (Semi-MsST-GAN). In the semi-supervised training process based on labeled and unlabeled images, for the data with ground truth, the multi-scale self-transformer network (MsSTNet) is trained to segment the corneal ulcer region as close to the corresponding ground truth as possible based on the guidance of the objective function of *L*_joint_. Then, the segmentation result of MsSTNet is concatenated with the original data and fed into the discriminator. At the same time, the ground truth is concatenated with the original data. They are all fed into the discriminator to discriminate whether the input pair is real or fake based on the objective function of *L*_D_. For the data without ground truth, MsSTNet is trained to segment the corneal ulcer region to confuse the discriminator to predict fake results based on the objective function of *L*_adv_. Then, the segmentation result of MsSTNet is concatenated with the original data and fed into the discriminator. The discriminator is trained to discriminate whether the input pair is real or fake based on the objective function of *L*_D_.

As shown in Figure 2, similar to general GAN methods (Mirza and Osindero, 2014; Isola et al., 2017), our proposed Semi-MsST-GAN mainly consists of two networks of generator and discriminator. The generator network aims to accurately segment the region of the lesion to confuse the discriminator, while the discriminator aims to discriminate whether its input paired is real or fake. It can be seen from Figure 2 that MsSTNet is employed as the generator of MsST-GAN. The Semi-MsST-GAN is trained based on the data composed of labeled images and unlabeled images:

(1) For the data with ground truth: MsSTNet is trained to segment the corneal ulcer region as close to the corresponding ground truth as possible based on the guidance of objective function of *L*_joint_. Then, the segmentation result of MsSTNet is concatenated with the original data (fake pair) and fed into the discriminator. At the same time, the ground truth is concatenated with the original data (real pair). They are all fed into the discriminator to discriminate whether the input pair is real or fake based on the objective function of *L*_D_.
(2) For the data without ground truth: MsSTNet is trained to segment the corneal ulcer region to confuse the discriminator to predict fake results based on the objective function of *L*_adv_. Then, the segmentation result of MsSTNet is concatenated with the original data and fed into the discriminator. The discriminator is trained to discriminate whether the input pair is real or fake based on the objective function of *L*_D_.

It should be noted that the optimization of Semi-MsST-GAN is an end-to-end training process based on mixed data composed of labeled data and unlabeled data.

#### Multi-Scale Self-Transformer Network

Recently, researchers have proposed several variant networks based on the encoder–decoder architecture for semantic segmentation tasks, such as SE-Net (Hu et al., 2018), CE-Net (Gu et al., 2019), Attention U-Net (Oktay et al., 2018), U-Net++ (Zhou et al., 2018), and CPFNet (Feng et al., 2020). Most of them introduced an attention mechanism to capture more rich details of objects instead of the direct concatenation of feature maps from the encoder and decoder. However, such attention-based feature extraction method still learns feature relationships in limited receptive fields, which cannot capture the long-range feature dependencies in the entire feature map.

**FIGURE 3:**
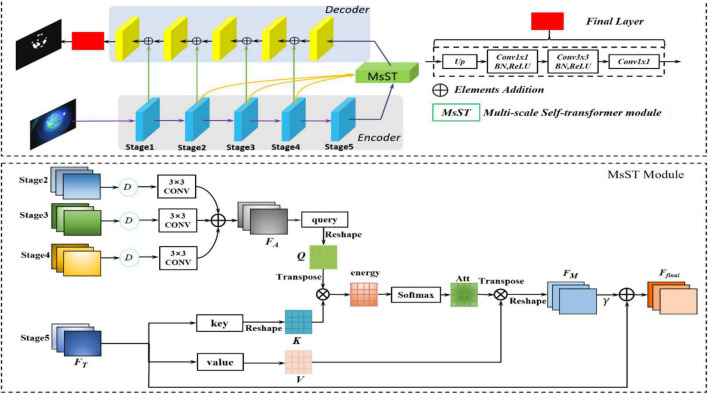
Overview of the proposed multi-scale self-transformer network (MsSTNet). The original image is fed into the encoder path composed of a pre-trained ResNet-18 to obtain the high-level features. Then, the feature maps from stages 2, 3, 4, 5 are fed into the MsST module to fuse multi-scale and multi-semantic information. Subsequently, the features are recovered by the decoder path. Finally, the predicted images are obtained.

In Lazebnik et al. (2006), Springenberg et al. (2014), He et al. (2015), and Long et al. (2015), contexts were encoded in the gradually larger receptive fields, which can model long-range dependencies. Long-range dependencies play a vital role in image analysis tasks based on deep neural networks (Fukushima and Miyake, 1982; LeCun et al., 1989; Yu and Koltun, 2015). Fukushima and Miyake (1982) and Yu and Koltun (2015) captured the long-range dependency features contained in the feature map by constructing a larger receptive field. LeCun et al. (1989) proposed a novel non-local neural network based on a self-attention mechanism to capture long-range dependencies. However, there is still the problem of non-local spatial interactions that are not cross scales (LeCun et al., 1989; Wang X. et al., 2018). Thus, these methods cannot capture the non-local context of objects with different scales (Zhang et al., 2020), especially for medical image segmentation tasks with complex pathological features (Chen et al., 2017; Zhao et al., 2017). Considering the loss of point-flaky mixed corneal ulcer in high-level feature maps resulting from the continuous downsampling operation, the feature maps from different levels were adopted to supplement long-range dependencies. Therefore, to fully utilize the feature interaction between the local context and the global context, which contains long-range dependencies and spatial correlations from different levels, we developed a novel MsSTNet as the segmentor of MsST-GAN. As shown in Figure 3, it adopts a pyramid architecture and self-attention layers to fuse feature maps cross spatial and scales. Figure 3 also shows that, in MsSTNet, the encoder–decoder architecture was also employed as our framework, in which the pre-trained ResNet-18 was adopted as the encoder path and simple upsampling and deconvolution constituted the decoder path. Especially, to reduce the semantic gap and avoid irrelevant clutters, a novel multi-scale self-transformer (MsST) module was proposed and embedded into the MsSTNet to enhance the ability of the model to extract multi-scale and multi-semantic features, which can improve the segmentation performance.

Figure 3 shows that the proposed novel MsST module was embedded into the top of the encoder path. Firstly, feature maps from stage 2 (*F*_1_), stage 3 (*F*_2_), and stage 4 (*F*_3_) were fed into a downsampling normalized module, which consists of a bilinear downsampling operation, followed by a 3 × 3 convolution layer to match the features of stage 5 in the channels and size. Then, the feature maps with different scales and semantic information were fused by the addition of elements. Finally, the fused feature maps with rich multi-scale and multi-semantic information and the feature maps of the top layer with global feature information were fed into self-attention (often called scaled-dot attention in natural language processing, NLP), which has three branches: query, key, and value (Shaw et al., 2018). As shown in Figure 3, to further extract rich features with complex pathological characteristics and suppress the interference from irrelevant features, we employed the fused feature maps with rich multi-scale and multi-semantic features as the input of branch query. The feature maps with rich global features, which are from the encoder’s top layer, were adopted as the input of branch key and value. In this way, it guides the model to learn salient global features and suppress the interference of unrelated local features. As can be seen from Figure 3, our proposed MsST module mainly consists of four steps:

(1) We adopted 1 × 1 convolution to encode the feature map *F*_A_ to query (*Q*) and encode *F*_T_ to key (*K*) and value (*V*), respectively.


(1) Q=Conv 1×1(FA)∈RB,C/8,W,H

(2) K=Conv 1×1(FT)∈RB,C/8,W,H

(3) V=Conv 1×1(FT)∈RB,C,W,H

(2) Calculate the similarity between query and key to obtain the non-local spatial feature correlation weight guided by global information. ° represents the pixel-wise multiple, as follows:


(4) $$Q=\text{Reshape}\left(Q\right)\in R^{B,C/8,W\times H}$$

(5) $$K=\text{Reshape}\left(K\right)\in R^{B,C/8,W\times H}$$

(6) energy=QTK°∈RB,W×H,W×H

(7) $$Att=\text{Softmax}\left(\text{energy}\right)\in R^{B,W\times H,W\times H}$$

3) The attention map Att and the corresponding *V* were weighted and summed to obtain the final spatial response *F*_M_ with a multi-scale and multi-semantic feature.


(8) FM=Reshape(VAttT°)∈RB,C,W,H

4) Finally, we multiplied *F*_M_ by a scale parameter, γ, and performed an element-wise summation operation with the feature map *F*_T_ to obtain the final output.


(9) $$F_{\text{final}}=F_{T}+\gamma \times F_{M}\in R^{B,C,W,H}$$

where γ is initialized as 0 and gradually learns to assign more weight. It can also be seen from Eq. 9 that the final feature map, *F*_final_, is the weighted sum of the multi-scale, multi-semantic, and strong semantic global features. Therefore, it not only has a global contextual view but can also selectively aggregate contextual information with multi-scale and multi-semantic features.

#### Discriminator

The ordinary GAN discriminator maps the input into a real number between 0 and 1, which represents the probability that the input sample is true or fake. It is not suitable for medical image segmentation, which requires high-resolution and high-definition details. Therefore, in this paper, the discriminator of patchGAN (Isola et al., 2017) was employed as the discriminator of MsST-GAN to solve these problems. It could classify whether each *N* × *N* patch from the input image is real or fake. This operation encourages the model to pay more attention to the structure in local patches, which is in favor of modeling high frequencies. The discriminator performs convolution operations on the input images, followed by averaging all responses to provide the ultimate discrimination of the output image. In this paper, *N* was set as 70.

### Loss Function

Given an input image ***X***, the segmentor and discriminator were denoted as MsSTNet and *D*, respectively. The segmentation results from MsSTNet were represented as MsSTNet (***X***). The input of *D* was defined as *X*_D_, which contains two forms: the original image combined with the ground truth (*X*_DT_) and the original image combined with the segmentation result (*X*_DF_), representing the pairs as True or Fake.

#### Loss for Discriminator

The spatial binary cross entropy loss *L*_D_, as follows, was adopted to optimize the discriminator:


(10) $$L_{\text{D}}=\sum_{h,w}\left(1-y)log(1-D{(\text{MsSTNET (X))}}^{h,w}\right)+y\log\left(D{\left(y\right)}^{h,w}\right)$$

where *y* = 0 if the patch was from MsSTNet prediction and *y* = 1 if the patch was from the ground truth. *D*(MsSTNet(***X***))*^h,w^* denotes the probability map of MsSTNet(***X***) at location (*h*,*w*), and *D*(*y*)*^h,w^* is the probability map of *y* at location (*h*,*w*).

#### Loss for MsSTNet

To improve the segmentation accuracy of MsSTNet, we proposed a novel joint loss function to optimize the model, as follows:


(11) $$L_{\text{joint}}=L_{\text{BCE}}+L_{\text{Dice}}+L_{\text{Adv}}$$

It can be seen from Eq. 11 that the joint loss function mainly contains three components: adversarial loss function, *L*_Adv_, which helps the segmentor generate prediction as close to the ground truth as possible; spatial cross entropy loss function, *L*_BCE_, which was mainly adopted to evaluate the gap between the segmentation result and the ground truth pixel-wise; and the dice loss, *L*_Dice_, which was employed to evaluate the segmentation performance in images.


(12) $$L_{\text{Adv}}=-\sum_{h,w}\log(D{(\text{MsSTNET (X))}}^{h,w})$$

(13) $$L_{\text{BCE}}=-\sum_{h,w}\left(1-y)\log{\left(1-\hat{y}\right)}^{h,w}+y\log(\hat{y}\right)$$

(14) $$L_{\text{Dice}}=1-\frac{2\left(y\cap \hat{y}\right)}{y\cup \hat{y}}$$

where $\hat{y}$ denotes the segmentation result of MsSTNet.

#### Objective Function for Semi-Supervised Learning

In semi-supervised learning, the loss function often contains two components: supervised loss and unsupervised loss. Supervised loss was adopted to optimize the model based on the data with ground truth. Unsupervised loss was employed to evaluate the segmentation results, optimizing the model to accurately segment the data without ground truth. In this paper, the supervised and unsupervised losses were defined as follows:


(15) $$L_{\text{supervised}}=L_{\text{joint}}+L_{\text{D}}$$

(16) $$L_{\text{unsuperivised}}=L_{\text{Adv}}$$

The semi-supervised loss function was finally defined as follows:


(17) $$L_{\text{semi}}=L_{\text{supervised}}+L_{\text{unsuperivised}}$$

## Dataset

To evaluate the performance of the proposed method, comprehensive experiments have been conducted on the SUSTech-SYSU public slit-lamp fluorescein staining image dataset (Deng et al., 2020), which was released to develop and evaluate automatic corneal ulcer segmentation algorithms. As far as we know, this is the first time the semi-supervised-based method has been explored for corneal ulcer segmentation task based on the SUSTech-SYSU dataset. It has 354 point-flaky mixed and flaky corneal ulcer slit-lamp fluorescein staining images with ground truth annotated pixel-wise by ophthalmologists and 358 point-like corneal ulcer images without ground truth, in which the lesions were too small to annotate. Each RGB image with a resolution of 2,592 × 1,728 pixels contains only one corneal area, which is located in the middle of the field of view. In order to achieve a balance between the computational efficiency and avoid the loss of lesions with small size, the original images and their ground truths were resized to 512 × 512 by bilinear interpolation. In order to fully demonstrate the effectiveness of our proposed method, the dataset was randomly divided into fourfolds. The data strategies are listed in Table 1 to train and evaluate all models. Besides, we also adopted online data augmentation, including rotations from −10 to 10 degrees, horizontal flipping, vertical flipping, Gaussian noise addition, and affine transformation to prevent overfitting and improve the robust ability of the model.

**TABLE 1:** Experimental data strategies.

Supervision approach	Data distribution
Supervised	All 354 labeled slit-lamp images were randomly divided into fourfold for cross-validation. Except for the 4th fold, which only had 84 images, each fold contained 90 slit-lamp images.
Semi-supervised	All 354 labeled slit-lamp images were randomly divided into fourfold for cross-validation. Except for the 4th fold, which only had 84 images, each fold contained 90 slit-lamp images. The 358 unlabeled point-like corneal ulcer images in the SUSTech-SYSU dataset were mixed with the labeled images to train the semi-supervised method.

## Experiments and Results

### Evaluation Metrics

To fully and fairly evaluate the segmentation performance of the different methods, four metrics were employed: dice coefficient (Dsc), Jaccard index (Jac), sensitivity (Sen), and Pearson’s product-moment correlation coefficient (PPMCC). PPMCC, with a value between −1 and 1, is often adopted to measure the correlation (linear correlation) between two variables. The four indicators were calculated as follows:


(18) $$\mathrm{Dsc}=\frac{2\times \text{TP}}{2\times \mathrm{TP}+\mathrm{TN}+\mathrm{FP}}$$

(19) $$\mathrm{Sen}=\frac{\text{TP}}{\mathrm{TP}+\mathrm{FN}}$$

(20) $$\mathrm{Acc}=\frac{\mathrm{TP}+\mathrm{FN}}{\mathrm{TP}+\mathrm{FP}+\mathrm{FN}}$$

(21) $$\mathrm{PPMCC}=\frac{\mathrm{Cov}\left(X,Y\right)}{\sigma_{X}\sigma_{Y}}$$

where TN, TP, FN, and FP represent true negative, true positive, false negative, and false positive, respectively. *X* and *Y* denote the segmentation result and corresponding ground truth, respectively. *Cov*(.) represents the covariance between *X* and *Y*. σ_*X*_ and σ_*Y*_ are the standard deviations of *X* and *Y*, respectively.

### Implementation Details

The proposed network was performed on the public platform Pytorch and a Tesla K40 GPU (12 GB). Adam was used as the optimizer. The initial learning rate was set to 0.0005, and weight decay was set to 0.0001. The batch size was set to be 4 and epoch was 100.

The segmentation performance of our proposed network was compared with other excellent networks, such as Attention U-Net (Oktay et al., 2018), R2U-Net (Alom et al., 2018), CE-Net (Gu et al., 2019), ResU-Net (He et al., 2016), PSPNet (Zhao et al., 2017), DeepLabv3+(Chen et al., 2018), U-Net++ (Zhou et al., 2018), and CPFNet (Feng et al., 2020). Aside from these CNN-based networks, the proposed network was also compared with other GANs, such as cGAN (Mirza and Osindero, 2014), PIX2PIX (Isola et al., 2017), and Cycle GAN (Zhu et al., 2017). Besides, several semi-supervised methods were also compared, such as Semi-cGAN, Semi-PIX2PIX, and Semi-Cycle GAN. All the networks were trained with the same parameters. It should be noted that all experiments based on supervised learning adopted the same data processing strategy and loss function of *L*_BCE_ + *L*_Dice_. Moreover, the code for Semi-MsST-GAN will be released in https://github.com/TingtingWang12/MsST-GAN.

### Experimental Results

Based on the data strategy listed in Table 1, we conducted comprehensive experiments to evaluate the effectiveness of our proposed MsST-GAN and Semi-MsST-GAN. MsST-GAN was compared with other CNN-based methods and GAN methods, with 354 labeled images under the supervised condition. Then, 358 unlabeled images were introduced to conduct the semi-supervised strategy. The proposed Semi-MsST-GAN was compared with Semi-cGAN, Semi-PIX2PIX, and Semi-Cycle GAN. Besides, we also conducted a series of ablation experiments to verify the validity of the proposed MsSTNet and loss function. For convenience, we used UNet (Ronneberger et al., 2015) as the baseline. The mean and standard deviation values of the four evaluation metrics and the efficiency for all methods are listed in Table 2.

**TABLE 2:** Evaluation indices for different methods.

Strategy	Methods	Dsc (%)	Sen (%)	Jac (%)	PPMCC (%)	Efficiency (s)
Supervised	U-Net (Ronneberger et al., 2015)	87.28 ± 5.38	88.54 ± 3.71	78.74 ± 8.13	87.40 ± 5.23	0.0015
	CE-Net (Gu et al., 2019)	88.43 ± 4.85	88.45 ± 4.31	80.38 ± 7.16	88.48 ± 4.53	0.0038
	Att-UNet (Oktay et al., 2018)	86.41 ± 6.17	88.05 ± 3.28	77.65 ± 9.05	86.59 ± 6.03	0.0026
	R2U-Net (Alom et al., 2018)	80.76 ± 9.26	82.56 ± 5.78	70.50 ± 11.71	81.29 ± 8.67	0.0042
	ResU-Net (He et al., 2016)	88.64 ± 4.73	89.02 ± 3.90	80.79 ± 7.33	88.71 ± 4.61	0.0029
	PSPNet (Zhao et al., 2017)	89.09 ± 4.64	90.20 ± 3.34	81.28 ± 7.25	89.08 ± 4.56	0.0030
	DeepLabv3+ (Chen et al., 2018)	88.29 ± 5.41	89.19 ± 4.90	80.32 ± 8.04	88.33 ± 5.27	0.0057
	U-Net++ (Zhou et al., 2018)	86.93 ± 4.66	87.31 ± 2.45	78.24 ± 6.97	87.05 ± 4.59	0.0022
	CPFNet (Feng et al., 2020)	89.38 ± 4.30	89.97 ± 2.50	81.76 ± 6.78	89.37 ± 4.23	0.0057
	cGAN (Mirza and Osindero, 2014)	85.22 ± 6.82	86.26 ± 3.37	75.25 ± 9.65	85.17 ± 6.51	0.0015
	PIX2PIX (Isola et al., 2017)	87.49 ± 5.31	87.81 ± 3.67	78.81 ± 7.92	87.55 ± 5.06	**0.0015**
	Cycle GAN (Zhu et al., 2017)	82.76 ± 9.40	80.35 ± 13.4	72.08 ± 13.28	82.98 ± 8.88	**0.0015**
Ablation supervised	Baseline (Ronneberger et al., 2015)	87.28 ± 5.38	88.54 ± 3.71	78.74 ± 8.13	87.40 ± 5.23	**0.0015**
	UNet+MsST	88.24 ± 4.63	90.03 ± 3.21	80.09 ± 7.20	87.85 ± 5.67	0.0022
	UNet+ResNet18	89.11 ± 4.56	90.02 ± 2.95	81.42 ± 7.08	89.11 ± 4.49	0.0021
	MsSTNet (UNet+ResNet18+MsST)	89.41 ± 4.36	90.04 ± 3.70	81.85 ± 6.87	89.41 ± 4.29	0.0025
	MsST-GAN (*L*_adv_ + *L*_D_)	89.21 ± 4.62	90.02 ± 2.98	81.36 ± 6.99	89.25 ± 4.37	0.0025
	MsST-GAN (*L*_adv_ + *L*_D_ *+ L*_BCE_)	89.31 ± 4.52	91.23 ± 2.39	81.44 ± 6.89	89.27 ± 4.33	0.0025
	MsST-GAN (L_adv_ + *L*_D_ *+ L*_Dice_)	89.64 ± 4.58	90.57 ± 2.75	82.11 ± 6.98	89.62 ± 4.38	0.0025
	**MsST-GAN**	89.90 ± 4.31	91.03 ± 1.88	82.36 ± 6.77	89.89 ± 4.12	0.0025
Semi-supervised	Semi-cGAN	83.87 ± 10.98	92.07 ± 4.40	73.89 ± 14.52	80.01 ± 18.07	**0.0015**
	Semi-PIX2PIX	87.28 ± 5.54	87.40 ± 4.11	78.58 ± 7.99	87.29 ± 5.34	**0.0015**
	Semi-Cycle GAN	82.35 ± 3.11	83.39 ± 6.87	70.79 ± 4.19	84.75 ± 5.71	**0.0015**
	**Semi-MsST-GAN**	**90.93** ± **4.19**	**91.93** ± **3.16**	**83.79** ± **6.72**	**90.77** ± **4.13**	0.0025

*Dsc, dice similarity coefficient; Sen, sensitivity; Jac, Jaccard index; PPMCC, Pearson’s product-moment correlation coefficient; cGAN, conditional generative adversarial network; MsSTNet, multi-scale self-transformer network; MsST-GAN, multi-scale self-transformer GAN. Values in bold indicate the best performance.*

It can be seen from Table 2 that both supervised MsSTNet and MsST-GAN outperformed other state-of-the-art supervised methods. Cycle GAN achieved the worst results with 82.76% for Dsc as it tended to model collapse, which may be caused by corneal ulcers with complex pathological features. Although the efficiency of our proposed MsST-GAN was slightly lower than that of the baseline (U-Net), the Dsc and Jac indices of MsST-GAN were improved by 3.00 and 4.60%, respectively, compared with U-Net. Moreover, compared with the latest excellent models such as CE-Net (Gu et al., 2019) and CPFNet (Feng et al., 2020), which have been adopted for various medical image segmentation tasks, the Dsc values of MsST-GAN were improved by 1.67 and 0.58%, respectively. Besides, the efficiency of the proposed method was also improved by 52 and 128% compared to CE-Net and CPFNet, respectively. These results show that our proposed method can improve the performance of segmenting corneal ulcers and satisfy real-time requirements by adopting non-local convolution and self-attention rather than the traditional attention mechanism.

**FIGURE 4:**
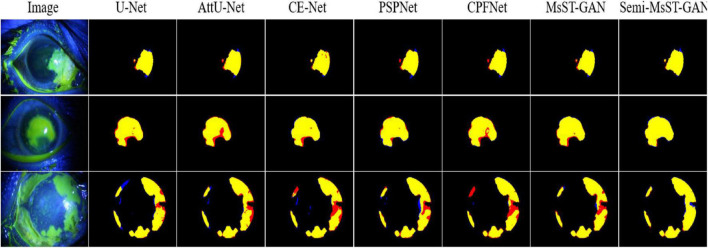
Examples of corneal ulcer segmentation. From *left* to *right*: original image, U-Net, Attention U-Net, CE-Net, PSPNet, CPFNet, MsST-GAN, and the proposed method. *Yellow* represents the correctly segmented region, while *red* and blue are the results of false-positive segmentation and false-negative segmentation, respectively.

The performance of our proposed Semi-MsST-GAN was further improved by introducing 358 unlabeled images obviously. Compared with MsST-GAN, the Dsc, Sen, Jac, and PPMCC of Semi-MsST-GAN were increased from 89.90, 91.03, 82.36, and 89.89% to 90.93, 91.93, 83.79, and 90.77%, by 1.03, 0.9, 1.43, and 0.88%, respectively. On the contrary, the evaluation metrics declined when cGAN and PIX2PIX introduced the semi-supervised strategy. It was mainly caused by the poor ability of cGAN and PIX2PIX to learn the complex pathological features of point-like lesions. These results show that the proposed Semi-MsST-GAN can improve the performance of segmentation by leveraging unlabeled images. Three examples of segmentation results with different methods are shown in Figure 4, where yellow represents the correctly segmented region while red and blue are the results of false-positive and false-negative segmentation, respectively. It can be seen from Figure 4 that our proposed method achieved the best segmentation results. The false-positive and false-negative segmentation results of the proposed Semi-MsST-GAN were obviously less than those of other methods. The results of U-Net (Ronneberger et al., 2015), Att-UNet (Oktay et al., 2018), CE-Net (Gu et al., 2019), and PSPNet (Zhao et al., 2017) had the problem of incorrect segmentation (shown in the bottom line of Figure 4). Compared with CE-Net (Gu et al., 2019), PSPNet (Zhao et al., 2017), and CPFNet (Feng et al., 2020), our proposed method cannot only accurately segment the lesion with small sizes but also maintain good regional continuity in segmenting large targets.

#### Statistical Significance Assessment

We further investigated the statistical significance of the performance improvement for the proposed MsST-GAN and Semi-MsST-GAN using the paired *t*-test. The *p*-values are listed in Tables 3, 4, respectively. To avoid confusion, we renamed MsST-GAN as “MsSTGAN” and Semi-MsST-GAN as “Semi MsSTGAN” in both tables. As shown in Table 3, compared with the other supervised learning-based methods, the proposed MsST-GAN achieved significant improvement in terms of the main evaluation metrics (Dsc and Jac), with *p*-values less than 0.05. Table 4 shows the *p*-values of the Semi-MsST-GAN compared with MsST-GAN and other CNN-based methods. All the improvements for the Jac and Dsc values of Semi-MsST-GAN were statistically significant, with *p* < 0.05, except for the Dsc of Cycle GAN (*p* = 0.052, slightly higher than 0.05). Tables 3, 4 further proved the effectiveness of the proposed MsST-GAN and Semi-MsST-GAN. Compared with those of the other CNN-based methods, the segmentation accuracies of both MsST-GAN and Semi-MsST-GAN have been significantly improved.

**TABLE 3:** Statistical analysis (*p*-value) of the proposed MsST-GAN compared with other convolutional neural network (CNN)-based methods.

Methods	Dsc	Jac
MsSTGAN–UNet (Ronneberger et al., 2015)	0.025	0.010
MsSTGAN–CENet (Gu et al., 2019)	0.040	0.008
MsSTGAN–Att-UNet (Oktay et al., 2018)	0.003	0.006
MsSTGAN–R2UNet (Alom et al., 2018)	0.038	0.036
MsSTGAN–ResUNet (He et al., 2016)	0.028	0.006
MsSTGAN–PSPNet (Zhao et al., 2017)	0.010	0.001
MsSTGAN–DeepLabv3+ (Chen et al., 2018)	0.014	0.014
MsSTGAN–UNet++ (Zhou et al., 2018)	0.015	0.008
MsSTGAN–CPFNet (Feng et al., 2020)	0.016	0.007
MsSTGAN–cGAN (Mirza and Osindero, 2014)	0.005	0.003
MsSTGAN–PIX2PIX (Isola et al., 2017)	0.005	0.001
MsSTGAN–Cycle GAN (Zhu et al., 2017)	0.049	0.045

*Dsc, dice coefficient; Jac, Jaccard index; cGAN, conditional generative adversarial network; MsST-GAN, multi-scale self-transformer GAN.*

**TABLE 4:** Statistical analysis (*p*-value) of the proposed Semi-MsST-GAN compared with MsST-GAN and other CNN-based methods.

Methods	Dsc	Jac
Semi MsSTGAN–UNet (Ronneberger et al., 2015)	0.013	0.026
Semi MsSTGAN–CENet (Gu et al., 2019)	0.016	0.017
Semi MsSTGAN–Att-UNet (Oktay et al., 2018)	0.005	0.001
Semi MsSTGAN–R2UNet (Alom et al., 2018)	0.043	0.020
Semi MsSTGAN–ResUNet (He et al., 2016)	0.010	0.017
Semi MsSTGAN–PSPNet (Zhao et al., 2017)	0.001	0.004
Semi MsSTGAN–DeepLabv3+ (Chen et al., 2018)	0.025	0.020
Semi MsSTGAN–UNet++ (Zhou et al., 2018)	0.025	0.026
Semi MsSTGAN–CPFNet (Feng et al., 2020)	0.006	0.010
Semi MsSTGAN–cGAN (Mirza and Osindero, 2014)	0.006	0.006
Semi MsSTGAN–PIX2PIX (Isola et al., 2017)	0.001	0.005
Semi MsSTGAN–Cycle GAN (Zhu et al., 2017)	0.052	0.043
Semi MsSTGAN–MsSTGAN	0.029	0.005
Semi MsSTGAN–Semi-cGAN	0.027	0.023
Semi MsSTGAN–Semi-PIX2PIX	0.001	0.001
Semi MsSTGAN–Semi-Cycle GAN	0.005	0.009

*Dsc, dice coefficient; Jac, Jaccard index; cGAN, conditional generative adversarial network; Semi MsSTGAN, semi-supervised multi-scale self-transformer GAN.*

#### Ablation Experiment for MsSTNet

As shown in Table 2, an ablation experiment was conducted to evaluate the proposed MsST module and the ResNet18 encoder path. Compared with the baseline model, our proposed MsSTNet (Baseline+MsST+ResNet18) achieved improvement in terms of all four evaluation metrics (2.13% for Dsc, 1.5% for Sen, 3.11% for Jac, and 2.01% for PPMCC). In order to demonstrate the performance improvement of the proposed MsST module and the ResNet18 encoder path, we also conducted the experiments of UNet+MsST and UNet+ResNet18. Compared with that of the baseline (UNet), the Dsc of UNet+MsST was improved from 87.28 to 88.24% and that of UNet+ResNet18 was improved from 87.28 to 89.11%, which benefits from the fact that the MsST module can guide the aggregation of low-level weak semantic information with the high-level strong semantic information and adaptively learn the spatial correlation in feature maps and the ResNet18 encoder path can extract feature effectively. These experimental results proved the effectiveness of the proposed MsST module and the ResNet18 encoder path.

#### Ablation Study for Loss Function

We also conducted experiments to demonstrate the effectiveness of our proposed loss function. It can be seen from Table 2 that, compared with MsST-GAN with only the generative adversarial loss function *L*_Adv_ + *L*_D_, both MsST-GAN with *L*_Adv_ + *L*_D_ *+ L*_BCE_ and with *L*_Adv_ + *L*_D_ *+ L*_Dice_ achieved higher values in all four evaluation metrics. Especially, the average Dsc of MsST-GAN with *L*_Adv_ + *L*_D_ *+ L*_BCE_ increased from 89.21 to 89.31%, while MsST-GAN with *L*_Adv_ + *L*_D_ *+* L_Dice_ increased from 89.21 to 89.64%. These results indicated that the effectiveness of *L*_BCE_ works at the pixel level and *L*_Dice_ works at the image level. Finally, the results of our proposed loss function *L*_supervised_ were compared with all the ablation experimental results. It can be seen from Table 2 that MsST-GAN with *L*_supervised_ achieved the best results in terms of Dsc, Acc, Jac, and PPMCC, except for Sen, which was slightly lower than that of the MsST-GAN with *L*_Adv_ + *L*_D_ *+ L*_BCE_. Especially, the Dsc and PPMCC of MsST-GAN with *L*_supervised_ were improved by 0.77 and 1.23% and reached 89.90 and 89.89% compared with the results of *L*_Adv_ + *L*_D_, respectively.

## Conclusion and Discussion

In this paper, we proposed a novel Semi-MsST-GAN for semi-supervised corneal ulcer segmentation, which mainly focused on solving two problems: (1) the interferences caused by large pathological differences between point-like, point-flaky, and flaky corneal ulcers, blurred boundary, and noise interference, and (2) how to improve the segmentation accuracy of the network by leveraging the data without ground truth. This is the first time the semi-supervision-based method has been introduced into the task of corneal ulcer segmentation, which achieved good results. Compared with other state-of-the-art supervised CNN-based methods, the newly proposed MsST-GAN achieved better segmentation performance with comparable efficiency. In addition, our proposed semi-supervision-based method can further improve the performance by leveraging the data without ground truth. Comprehensive experiments have been conducted to evaluate the effectiveness and robustness of the proposed method. The experimental results showed that, compared with that of the other state-of-the-art algorithms, the segmentation performance of our proposed semi-supervision-based method has been improved obviously.

There is still a limitation in this study. All the compared algorithms and the proposed semi-supervision-based method were trained and evaluated based on the limited data from the SUSTech-SYSU dataset. Although the proposed semi-supervision method has achieved better performance, we believe that if more data can be collected, the performance of the proposed method will be further improved. Therefore, it is one of our future works to collect more data and further improve the accuracy of segmentation.

## Data Availability Statement

The datasets presented in this study can be found in online repositories. The names of the repository/repositories and accession number(s) can be found below: https://github.com/CRazorback/The-SUSTech-SYSU-dataset-for-automatically-segmenting-and-classifying-corneal-ulcers.

## Ethics Statement

The studies involving human participants were reviewed and approved by the Zhongshan Ophthalmic Centre ethics committee of Sun Yat-sen University. The patients/participants provided their written informed consent to participate in this study. Written informed consent was obtained from the individual(s) for the publication of any potentially identifiable images or data included in this article.

## Author Contributions

TW conceptualized and designed the study, wrote the first draft of the manuscript, and performed data analysis. MW, WZ, LW, ZC, YP, FS, YZ, CY, and XC performed the experiments, collected, and analyzed the data. All authors contributed to the article and approved the submitted version.

## Conflict of Interest

The authors declare that the research was conducted in the absence of any commercial or financial relationships that could be construed as a potential conflict of interest.

## Publisher’s Note

All claims expressed in this article are solely those of the authors and do not necessarily represent those of their affiliated organizations, or those of the publisher, the editors and the reviewers. Any product that may be evaluated in this article, or claim that may be made by its manufacturer, is not guaranteed or endorsed by the publisher.
